# Supplementary material for: A 34.5 day quasi-periodic oscillation in γ-ray emission from the blazar PKS 2247–131
Source: Nat Commun. 2018 Nov 2;9:4599. doi: 10.1038/s41467-018-07103-2 (PMC6214961; doi:10.1038/s41467-018-07103-2)
Supplement: Supplementary file 1 — Supplementary Information [file 41467_2018_7103_MOESM1_ESM.pdf]

A 34.5 day quasi-periodic oscillation in γ-ray emission from the blazar PKS 2247−131

Zhou et al.

1. Supplementary Figures

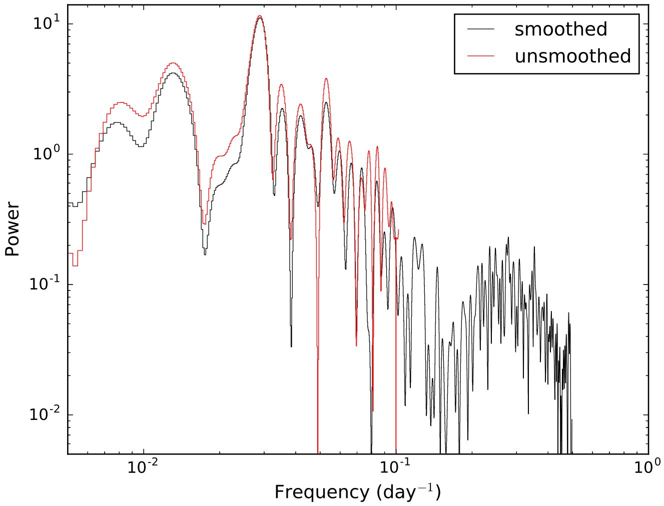

Supplementary Figure 1.— Comparison between the two power density spectra. The red and black curves are obtained from the 5-day binned light curve and the smooth light curve, respectively.

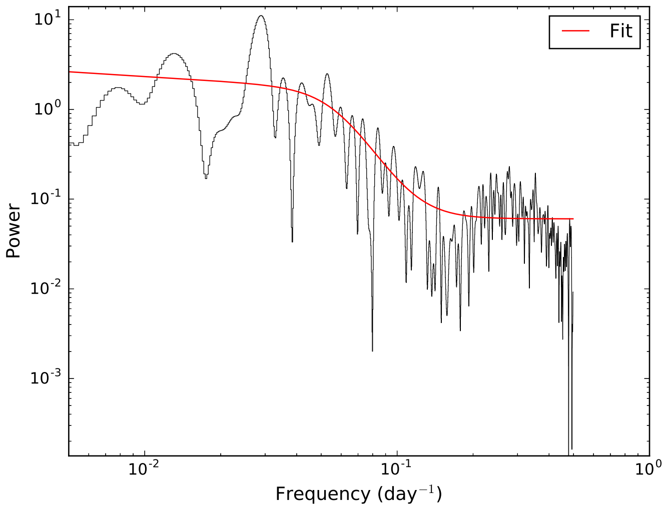

Supplementary Figure 2.— Model curve (red curve) of a smoothly bending power law. The curve is obtained by fitting the smoothed PDS (black curve).

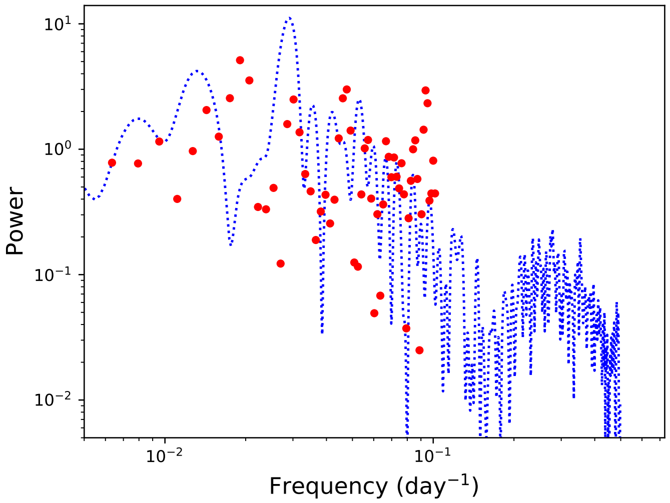

Supplementary Figure 3.— Example of the PDS data points (red dots). The data points result from one simulated light curve. The blue dashed curve is the original smoothed PDS.

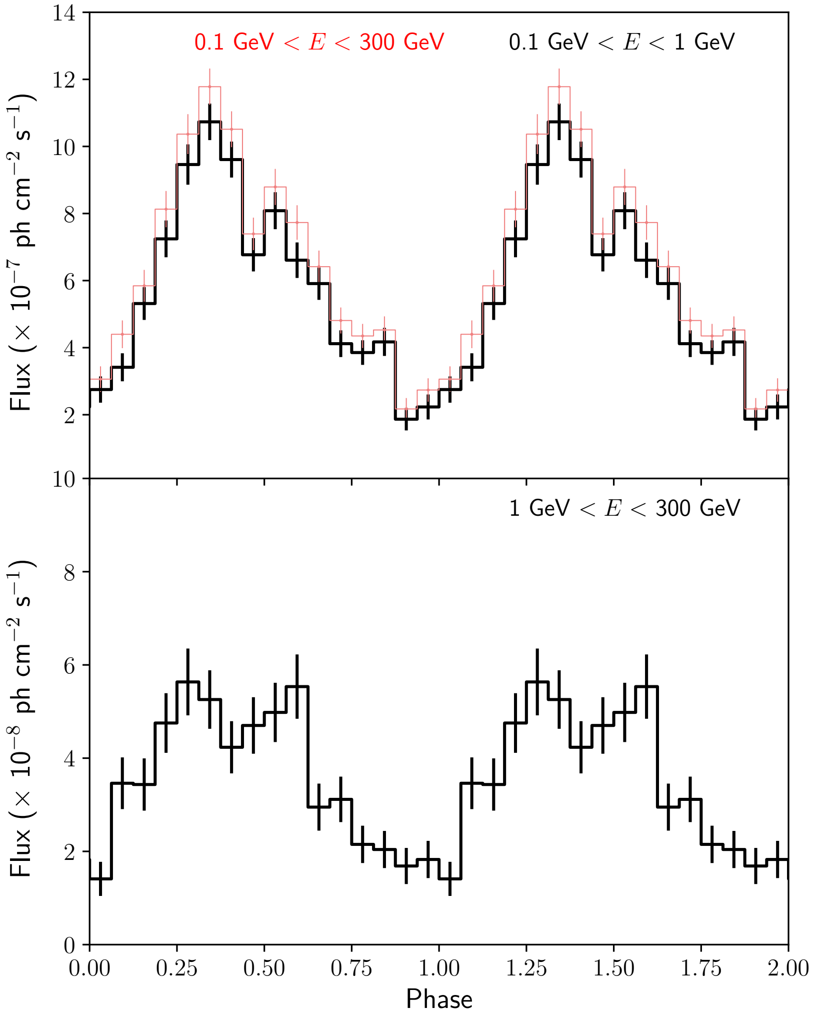

Supplementary Figure 4.— Folded light curves in the energy bands of < 1 GeV and > 1 GeV. They are constructed from binned likelihood analysis, where phase zero corresponds to MJD 57692.66. The error bars indicate 1σ uncertainties. In the top panel, the folded light curve obtained from the data of the full energy range 0.1–300 GeV is plotted for comparison (red histogram). In the high energy range of >1 GeV, shown in the bottom panel, the second component actually has a flux peak nearly as high as that of the main one.

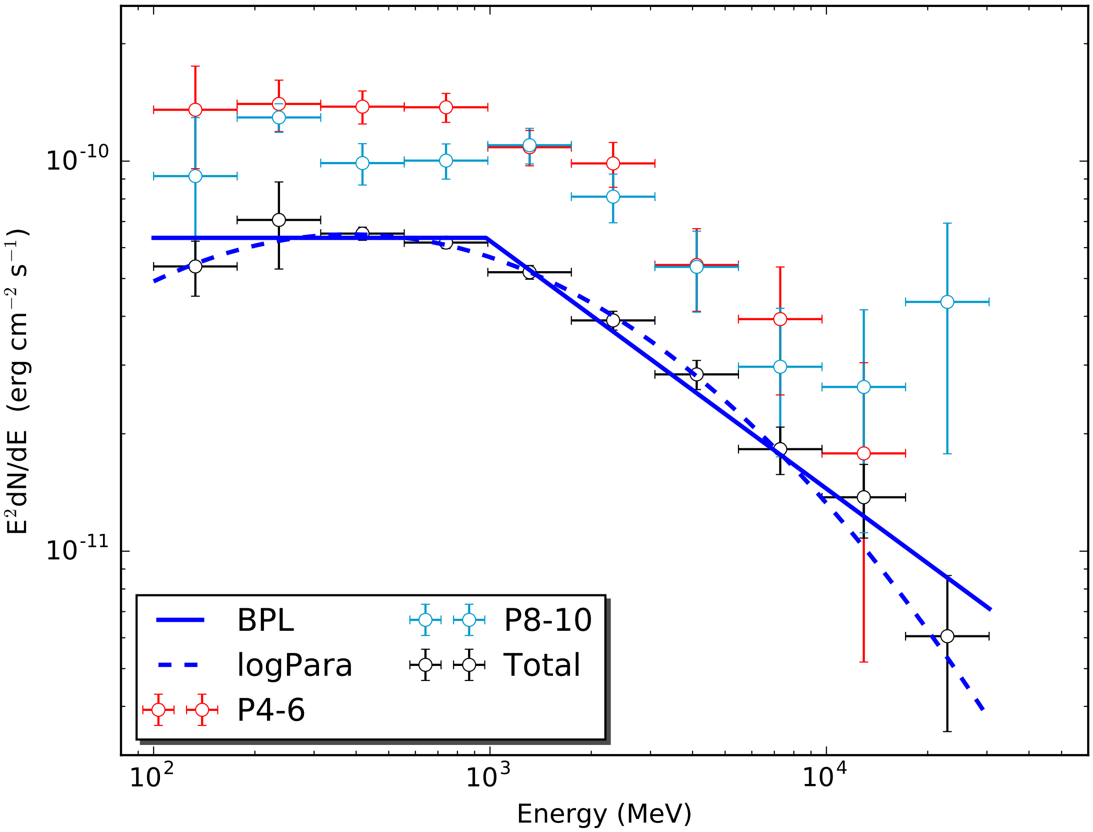

Supplementary Figure 5.— γ-ray Spectra of PKS 2247−131. The error bars are 1σ flux uncertainties. The black, and red and light blue data points are obtained from the total data, and the data in two phase ranges of 0.1875–0.375 and 0.4375–0.625, respectively. The latter two phase ranges are read from Supplementary Figure 4, covering the two peaks of the main and second components (see the main text). The spectrum from the total data is equally well described by a broken power law (blue solid line) or a log-parabola (blue dashed curve). From the comparison of the spectra, the second component has slightly lower fluxes in the < 1 GeV energy range.

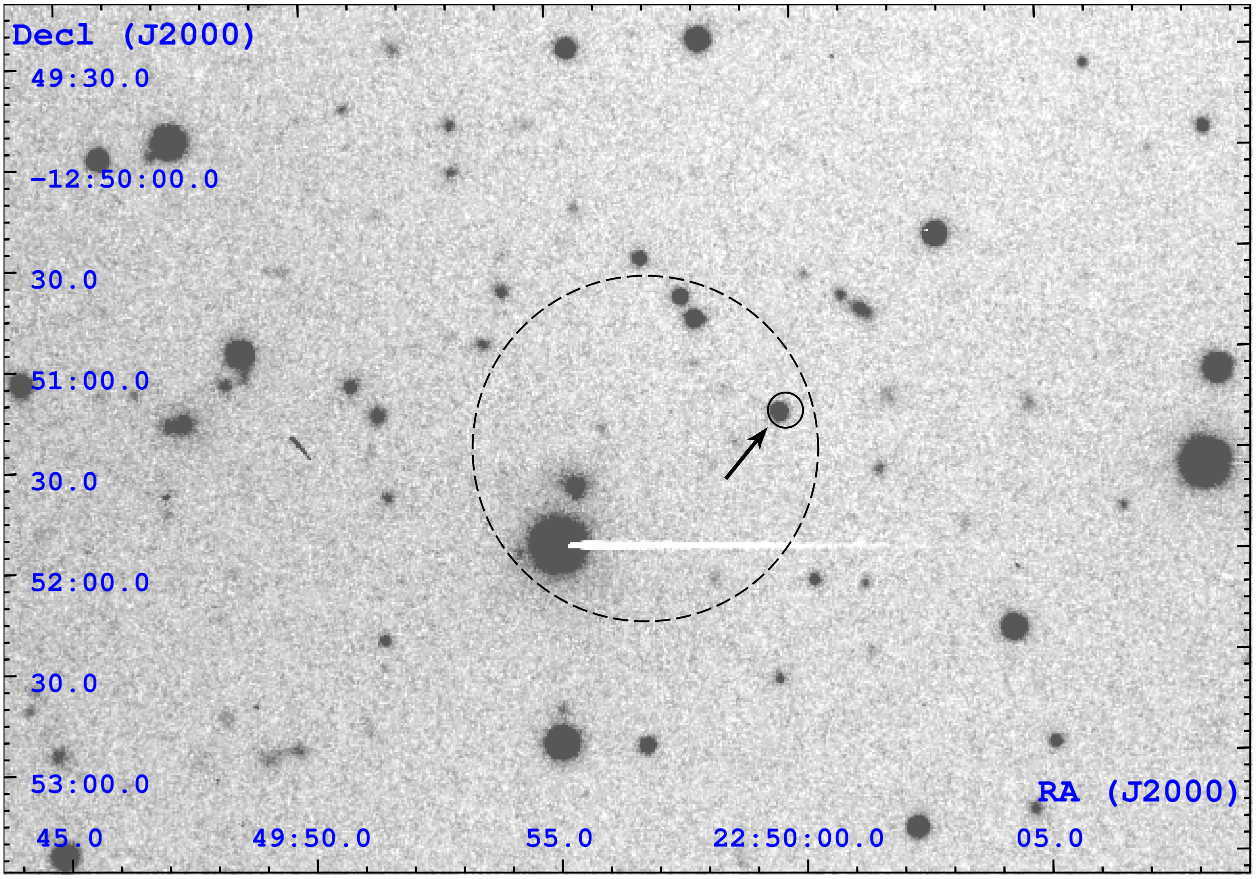

Supplementary Figure 6.— Optical V-band image of the PKS 2247−131 field. The image is made from the data taken with 2.4-m telescope at LiJiang Observatory on 2017 November 19. The imaging instrument was the Yunnan Faint Object Spectrograph and Camera, and the exposure time was 300 sec. The optical counterpart of PKS 2247−131, previously determined from radio and optical/infrared surveys, is marked by an arrow. The position of the X-ray source detected with *Swift* XRT is marked by a black solid circle, which well matches the optical counterpart. The large dashed circle marks the position, RA=$22^h49^m56^s.9$, Decl.=$-12°51'27''$ (equinox J2000.0; 1σ uncertainty $52''$), determined from the *Fermi* LAT data.

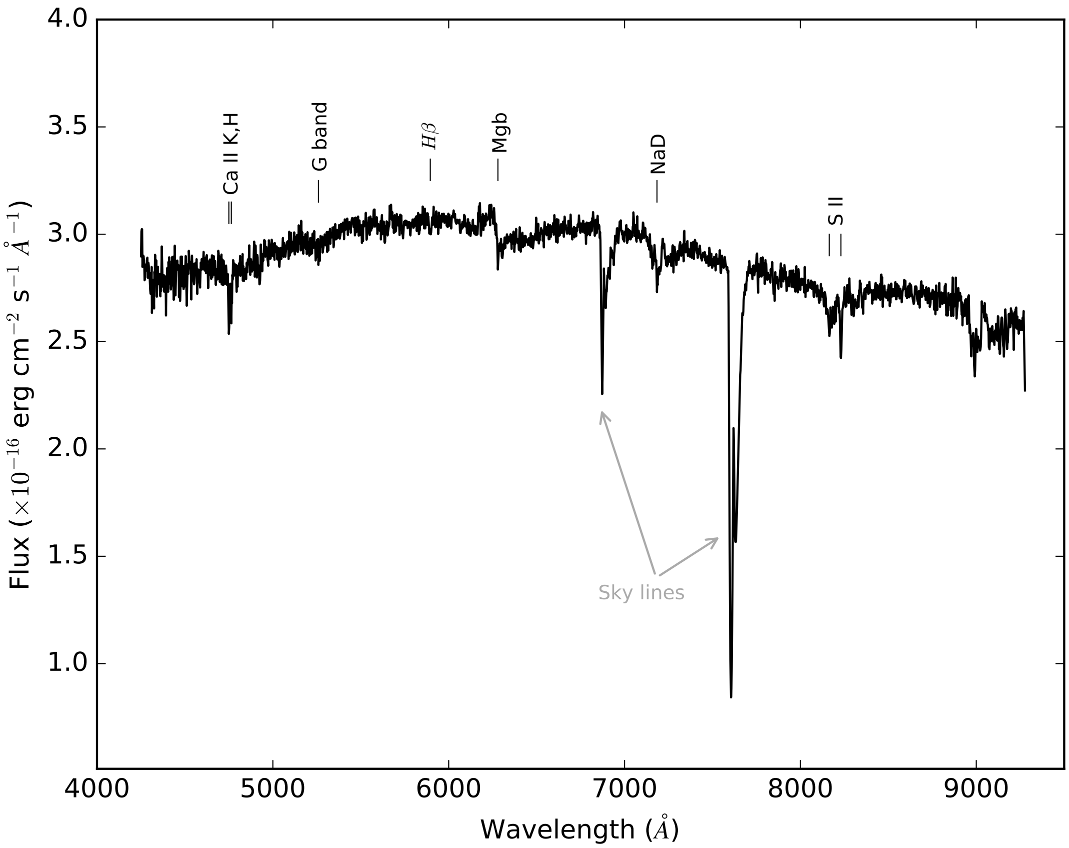

Supplementary Figure 7.— LDSS3 spectrum of the optical counterpart to PKS 2247−131. The observation was made during the outburst on 2017 November 22. The source spectrum is basically featureless, but a few weak absorption lines from the host galaxy were likely detected (approximately 3–10σ detections). Based on the absorption lines, a redshift of $z \approx 0.22$ is determined.

2. Supplementary Tables

Supplementary Table 1: Parameters for fits to the folded light curve

	A	μ	λ	$\chi^2/d.o.f.$
Lor_1	1.31±0.19	0.32±0.01	0.15±0.02	
Lor_2	0.89±0.23	0.60±0.02	0.19±0.04	24.4/10
Const.	0.95±0.46			

Note: Two Lorentz functions plus a constant are fit to the light curve. A Lorentz function is $\mathrm{Lor} = A\lambda/[\lambda^2 + (x-\mu)^2]$, where A is the amplitude, 2λ and μ are the full width at half maximum and centroid, respectively.

Supplementary Table 2: *Swift*-XRT observations of PKS 2247-131

ObsID	Date	Exposure time (s)	Count Rate (10^{-2}ct s^{-1})
00034636001	2016 Jul 31	2990	0.64 ± 0.17
00034636002	2016 Aug 03	990	< 2.10
00034636003	2016 Aug 04	276	< 4.00
00034636004	2016 Aug 05	1080	< 0.90
00034636005	2016 Aug 10	456	< 3.20
00034636006	2016 Aug 11	583	< 2.80
00034636007	2016 Oct 14	2961	0.92 ± 0.18
00034636008	2016 Oct 16	2261	1.02 ± 0.22
00034636009	2016 Oct 19	1196	0.92 ± 0.35
00034636011	2016 Oct 20	2673	1.09 ± 0.21
00034636013	2016 Oct 21	2987	0.84 ± 0.18
00034636014	2016 Oct 22	1397	1.44 ± 0.32
00034636017	2016 Oct 23	2625	1.19 ± 0.22
00034636019	2016 Oct 24	2529	1.51 ± 0.25
00034636020	2017 Dec 20	2989	1.34 ± 0.22
00034636021	2017 Dec 26	2636	1.10 ± 0.21

Note. — (1) Count rate in the energy range 0.3–10 keV.

Supplementary Table 3: Other flaring *Fermi* blazar sources

Source name	$F_{peak} \times 10^{-7}$ (ph cm^{-2} s^{-1})	Duration (days)
3FGL J0108.7+0134 (4C 01.02)	12.85 ± 0.21	540
3FGL J0238.6+1636 (AO 0235+16)	8.83 ± 0.16	270
3FGL J0403.9−3604 (PKS 0402−362)	6.13 ± 0.15	540
3FGL J0532.0−4827 (PMN J0531−4827)	4.14 ± 0.11	540
3FGL J0841.4+7053 (S5 0836+71)	8.34 ± 1.16	450
3FGL J1048.4+7144 (S5 1044+71)	7.46 ± 0.15	540
3FGL J1104.4+3812 (Mrk 421)	4.74 ± 0.11	540
3FGL J1127.0−1857 (PKS 1124−186)	3.62 ± 0.12	540
3FGL J1146.8+3958 (S4 1144+40)	3.53 ± 0.13	630
3FGL J1159.5+2914 (Ton 599)	10.50 ± 0.17	270
3FGL J1224.9+2122 (4C 21.35)	26.47 ± 0.27	630
3FGL J1229.1+0202 (3C 273)	18.46 ± 0.28	450
3FGL J1239.5+0443 (MG1 J123931+0443)	6.91 ± 0.17	270
3FGL J1256.1−0547 (3C 279)	17.84 ± 0.24	180
3FGL J1332.0−0508 (PKS 1329−049)	6.26 ± 0.20	540
3FGL J1427.9−4206 (PKS B1424−418)	16.62 ± 0.23	450
3FGL J1504.4+1029 (PKS 1502+106)	12.47 ± 0.18	450
3FGL J1512.8−0906 (PKS 1510−08)	26.66 ± 0.30	270
3FGL J1522.1+3144 (B2 1520+31)	5.81 ± 0.13	630

Notes. The 19 blazars found to have fluxes and flaring durations comparable with those of the flare seen in PKS 2247−131. The durations are estimated from 3-month binned light curves of the blazars.

3. Supplementary Methods

The frequency range of a power density spectrum (PDS) is set by the total time length (210 days for our data from MJD 57693 to 57903) and time bin of a light curve. In our case, in order to well describe the white noise of the PDS, we used the smooth light curve (that was constructed from shifting 5-day binned data by only one day forward) to obtain a "smoothed" PDS. This way, the 5-times better resolution of the smooth light curve allowed for the extension of the high-frequency end by a factor of 5. The comparison of the two power density spectra, obtained from the 5-day binned light curve and the smooth light curve, is shown in Supplementary Figure 1, where the latter PDS is lowered by a factor of 5 for the comparison. As can be seen, at the low frequency range of < 0.1 day^{-1}, the two spectra match well.

We excluded the ∼34.5-day signal from the smoothed PDS, and fit the rest of the PDS with a smoothly bending power-law model (see Equation 2 in Reference 1 for details) $P(f) = Af^{-\alpha_{\rm low}}/[1 + (f/f_{\rm bend})^{\alpha_{\rm high}-\alpha_{\rm low}}] + c$. A maximum likelihood method was used to determine the parameters in this model, since the fitted data are not Gaussian distributed.[2] We obtained normalization $\log_{10}A = -5.92 \pm 0.10$, bend frequency $\log_{10}f_{\rm bend} = -1.23 \pm 0.02$, Poisson noise level $\log_{10} c = -1.22 \pm 0.01$, high-frequency slope $\alpha_{\rm high} = 0.18 \pm 0.17$, and low-frequency slope $\alpha_{\rm low} = 4.99 \pm 0.05$. The obtained model curve, compared to the PDS, is shown in Supplementary Figure 2.

Using the model curve with the Python code of the Emmanoulopoulos light curve simulation algorithm,[3] we generated a total of 10^7 simulated light curves. For each light curve, we obtained its PDS data points. An example of the PDS data points from one simulated light curve is shown in Supplementary Figure 3. The significance at a frequency was estimated by counting the data points over a significance level (e.g., at a 5σ level at a frequency, there are only 6 data points out of 10^7). The significance curves were thus obtained, which were used to find a significance of 5.2σ for the 34.5-day signal. The independent trials were 20, as the data of the 210-day time length were binned into 42 5-day bins, and the number of independent frequencies up to the Nyquist frequency was $42/2 - 1 = 20$ (e.g., see Reference 4). After considering this trial number, the significance was lowered to 4.6σ.

Supplementary References

1. Emmanoulopoulos, D., McHardy, I. M., & Papadakis, I. E. Generating artificial light curves: revisited and updated. *Mon. Not. R. Astron. Soc.* **433**, 907–927 (2013).

2. Connolly, S. A Python Code for the Emmanoulopoulos et al. [arXiv:1305.0304] Light

Curve Simulation Algorithm. Preprint at https://arXiv.org/abs/1503.06676 (2015).

3. Barret, D., & Vaughan, S. Maximum Likelihood Fitting of X-Ray Power Density Spectra: Application to High-frequency Quasi-periodic Oscillations from the Neutron Star X-Ray Binary 4U1608-522. *Astrophys. J.* **746**, 131 (2012).

4. van der Klis, M. Fourier techniques in X-ray timing. in Timing Neutron Stars, ed. H. Ogelman, E.P.J. van den Heuvel (Dordrecht: Kluwer), NATO ASI Series C, **262**, 27 (1989).

This preprint was prepared with the AAS LaTeX macros v5.0.
